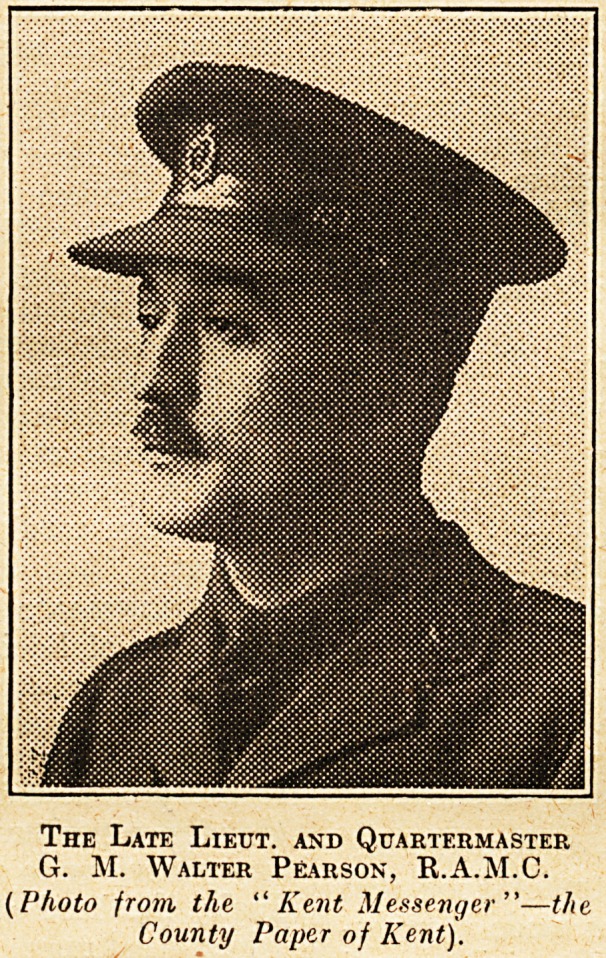# Hospital and Institutional News

**Published:** 1918-06-15

**Authors:** 


### HOSPITAL AND INSTITUTIONAL NEWS.

### A TESTIMONIAL TO DR. RHODES.

WE are glad to see that the whole body of the establishment governors of North Staffordshire Infirmary have decided to give a testimonial to Dr. Basil Rhodes, the medical superintendent, on his retirement to take up important work elsewhere. The Rev. Prebendary H. V. Stuart remarked that the testimonial would be not so much to the work accomplished as to the spirit in which it had been carried out. Dr. Rhodes was not the efficient official—a type which is apt to be both officious and offensive. He was always ready to listen to suggestions, and met men and women from every quarter in the spirit which has given the infirmary a sure hold upon the affections of all classes of people. The spirit which had maintained this affection was that which they wished to mark. For no money could buy it, and no money could reward it either. Sir Henry Burdett's recent visit had taken place some eleven years since he inspected and reported upon the hospital. Those who remembered his criticisms then would appreciate the changes which had lately received praise from the same quarter. These changes were largely due to the personality of Dr. Rhodes. A subcommittee was appointed, and the representatives of the various works are to take what steps seem proper to them. The Hospital Saturday Committees have been asked to co-operate. The money subscribed it was resolved to place in the hands of the President not later than June 14 (*i.e.* within fourteen days), so that the presentation may be made to Dr. Rhodes before his departure on June 20.

### HOSPITAL SECRETARY'S DEATH IN MESOPOTAMIA.

WE regret to record the death of Lieut. and Quartermaster G. M. Walter Pearson, R.A.M.C., who, until his enlistment in November 1915, was secretary of Gravesend Hospital. He is reported to have died of burns in Mesopotamia, where he was attached to a Field Ambulance. Walter Pearson—for those, like the present writer, who were personally acquainted with him will always think of him as a civilian—began his responsible hospital life at Buxton, where he became assistant secretary to the Devonshire Hospital in 1902. In 1908 he was appointed to the secretaryship of the Gravesend Hospital, where he showed his keenness in many successful organisations. His collectors and helpers were always sure of his leadership, and he was not content to measure his success or theirs merely by the higher totals which these organisations used to show year after year. His success lay in making the hospital a centre of local interest and in creating a personal tie between it and most of the local residents. While quietly at work at Gravesend he had his ambitions, which were hardly apparent to those who had not the clue to his genial and easy-going manner. We recall a long conversation with him on the difficulties and slowness of promotion in hospital life, and how he compared, with a sense rather of amusement than of annoyance, the salaries obtainable in commercial life to men with responsibilities equal to those of a hospital secretary. On his enlistment in November 1915 he became Lieut. and Quartermaster in the Royal Army Medical Corps. In February of the following year he married, and in less than a fortnight had started for Mesopotamia. He remained there till his death on May 24. There is little doubt that, had his life been spared, he would soon have gained promotion. Indeed, he narrowly escaped appointment to the secretaryship of a big Midland hospital just before the war. Members of the Hospital Officers' Association will remember him for his geniality and for his promise. We respectfully offer our sympathy to Mrs. Pearson, who is nursing at a hospital at Leicester. It is remarkable that Mr. A. E. Thomas, his predecessor, was killed in France in November 1914. We hope that the Gravesend Hospital will do its duty to his memory, for it owes much to his work. The present acting secretary of the Gravesend Hospital is Mr. C. E. Chapman.

THE LATE LIEUT. AND QUARTERMASTER G. M. WALTER PEARSON, R.A.M.C.
(*Photo from the "Kent Messenger"—the County Paper of Kent*).

### THE NEW SUPERINTENDENT OF THE DEVONSHIRE HOSPITAL, BUXTON.

MR. TOM B. HARRISON, secretary to the Royal West Sussex Hospital, Chichester, has been appointed general superintendent and secretary of the Devonshire Hospital, Buxton. Mr. Harrison, who is thirty-five years of age, has been engaged in hospital work since 1896, when he became clerk to the North Staffordshire Infirmary. In 1900 he was promoted senior clerk, a post which he retained till 1905, when he joined Mr. R. J. Coles' staff at Addenbrooke's Hospital, Cambridge. Under that able administrator Mr. Harrison widened his experience and learnt new methods, for Mr. Coles was always fertile and persistent. He remained at Cambridge for five years, when he was appointed secretary of the Chichester Infirmary. We emphasise the old title of that hospital because it was only some time after Mr. Harrison's arrival in 1910 that it launched the reconstruction scheme, the success of which led to its official recognition under the name of the Royal West Sussex Hospital, Chichester. Mr. Harrison was, if we remember, the first paid professional secretary whom the Chichester institution appointed. His appointment was the outcome of a desire to conduct the reconstruction scheme successfully, and Mr. Harrison arrived fresh from the improvements at Cambridge, on which Mr. Coles, with his help, had been engaged. At Chichester he had two main tasks to perform to carry out the reconstruction scheme, and to reorganise the income, which had fallen short of the expenditure for a considerable time. In both success was gained. Mr. Harrison, who is married and has three children, is therefore well equipped for his new post.

### THE PRINCE OF WALES' BIRTHDAY.

JUNE 23 is the birthday of the Prince of Wales, who is the President of the Westminster Hospital. The institution is anxious to celebrate the occasion by announcing a substantial contribution towards the Special Appeal Fund. It therefore invites all who possess the Christian name of Edward to send at least one shilling to the fund. The committee needs £20,000. There must be sufficient Edwards in London alone to raise the bulk of this sum before the Prince of Wales' birthday. Perhaps Sir Edward Carson will set the ball rolling.

### A POTTED REPORT.

THE committee of Addenbrooke's Hospital, Cambridge, has decided to economise paper and avoid expense. The report of the hospital, therefore, has shrunk to a four-page leaflet, which contains this decision on the first page, the revenue account within, and nothing on the back leaf. This is the shortest report which we have seen since the war. The committee's report and the accounts are, of course, on view at the secretary's office.

### REDUCTION OF THE HOSPITAL JAM RATION.

WE are informed by the Ministry of Food that the present position of supplies has made it necessary to reduce the jam ration in the civil general hospitals from eight ounces to four ounces. Institutions entitled to this scale should make application to the Jam Section, Ministry of Food, 100 Cromwell Road, S.W. 7, for the average quantity required per week on the basis of a ration of four ounces. Arrangements will then be made for supplies to be forthcoming, though no absolute guarantee can be given.

### ORDER OF THE BRITISH EMPIRE.

ON the occasion of His Majesty's birthday a large number of promotions in and appointments to the Most Excellent Order of the British Empire have been made for services in connection with the war. The first instalment of the list appeared in the *Times* of June 8, and further instalments have appeared in that journal daily up to and including June 13. The list includes members of the Council of the British Red Cross Society and of the Joint War Committee; heads of and workers in various departments of the British Red Cross Society; Red Cross Commissioners; County Directors of auxiliary hospitals and V.A.D.s; donors and administrators of hospitals; organisers of Hospital Supply Depôts; V.A.D. Directors, and others. The names of several medical men appear in the list, including that of Dr. John Lumsden, Vice-Chairman of Joint V.A.D. Committee, Ireland, who becomes a Knight Commander of the Order. Miss Agnes Weston, founder of the Royal Sailors' Rests at naval ports, becomes a Dame Grand Cross.

### A SCHOOL OF ARMY SANITATION.

IN the grounds of Beckett's Park Hospital, at Leeds, an institution known as a School of Army Sanitation has been established to investigate and make known the best way to prevent and minimise such diseases as "trench fever," bilharziasis, an intestinal ailment which occurs in Egypt, "Bagdad sore," and similar diseases of warfare. Instruction in sanitation is given by a series of exhibits, diagrammatic pictures, and models. How to serve food to troops in different climates and conditions, how to disinfect clothes and destroy vermin, sterilise water, and get rid of refuse. One exhibit deals with vaccines, another shows how to catch flies by the bucketful. The school, in fact, has an Eastern section in which the means of destroying insects are illustrated. Many of the models have been made by the wounded in the curative workshops, and the school has grown from a set of instructions designed for American officers into its present more permanent and ambitious form. The medical officer in charge is Captain Daukes.

### A FRENCHMAN'S DISCOVERY IN ORTHOPÆDICS.

A BATH of liquid paraffin wax has been found a useful preparation for massage at the 2nd Northern General Hospital, Beckett's Park, Leeds. Enthusiasts say that such a bath ought to be added to every orthopædic centre, but it was by chance that the experiment was first made. Seven years ago, according to a published statement, Mr. W. L. Ingle, the head of a firm of tanners and curriers which used much paraffin wax in the course of its business, heard that a Frenchman had started a liquid paraffin wax bath for the treatment of rheumatism. With the means at hand Mr. Ingle started a similar bath at his works, where, we understand, hundreds of cases were treated with success free of charge. On the opening of the orthopædic department at Beckett's Park Hospital Mr. Ingle informed Colonel Littlewood of his experience, which was put to the further test by the experiment of sending a batch of hospital patients to the works. It was found that, after the bath, pain in injured limbs was much relieved, and if the temperature was further raised and the bath prolonged to two or three hours massage was made easy because of the flexibility imparted to the limb. The bath, a steam-heated tank which holds a ton and a half of wax, was removed, at Mr. Ingle's offer, to the hospital, and 400 patients a week have been treated in the three months since the transfer took place.

### THE COMING GENERATION IN GERMANY.

Some interesting figures have been published by a commission appointed by the German Government to consider measures necessary to increase the population after the war. The first three years of hostilities have reduced by more than two million the number of babies who should have been born under normal conditions. Thus in 1916, as compared with 1913, there was a 40 per cent. reduction, whilst the infantile death-rate rose very early to an abnormally high figure. Special measures were speedily adopted to cope with this alarming increase, and at present the rate approximates to the pre-war figure of 151 per 1,000, though this, according to English standards, would be reckoned far too high. The committee suggest that marriage should be made compulsory at the age of twenty, and those failing to marry, or who are childless, should be penalised. Judging by the illegitimate birth-rate (10 per cent. of total births), the plural marriages scheme seems to have caught on, the patriotic Teuton being ever willing to sacrifice morality to patriotism.

### CANADIAN WAR CONTINGENT ASSOCIATION.

This association, whose annual meeting was held last month under the chairmanship of Sir G. Perley, the High Commissioner and its president, exists to supply a most miscellaneous assortment of comforts, luxuries, and necessities to the soldiers from Canada. Any officer commanding can requisition on the association for anything, and it is sent him, if possible, for his distribution. Maple sugar by the ton, candles, soap, paraffin, socks are some of the articles sent out. The receipts for the year have been over £20,000, and the expenditure £1,000 less than the receipts; but this balance in hand is not much, since the demands on the association are increasing, and the past year was marked by at least one substantial and non-recurrent gift—£4,000 that resulted from an appeal to the city of Glasgow. Money is still greatly needed. It is claimed that the 400,000 odd pairs of socks sent out practically prevented cases of trench feet. It is, however, probable that the good discipline of the Canadians, their obedience to and care in following the directions given them as to rubbing feet, changing, avoidance of pressure from tight puttees, and the watchful care of their officers for the welfare of their men had at least as much to say to the absence of this preventable trouble as the helpful supply of extra socks. We wish the best of luck to the association and to the brave men whose needs it serves.

### PLUCK AND GRIT.

On another page will be found an account of an operation for strangulated hernia. Beyond the interest of the medical aspect of the case, and of the surgical skill recorded, is the interest in the courage of the old lady who submitted herself to the operation, though inwardly convinced it was in vain, and who, finding she had recovered, despite her more than four-score years was eager at the earliest date to take up again laborious duties. We feel sure her stock are not conscientious objectors.

### PENSIONERS' WARDS—WHICH ONCE WERE EMPTY.

The David Lewis Northern Hospital, Liverpool, has opened a ward that has long been "closed for want of funds" and placed it at the disposal of the Ministry of Pensions. It contains twenty-eight beds, and will be called the Pensioners' Ward. Meanwhile an orthopædic department is being organised for out-patients. It is always a relief to see a closed ward reopened, and now that the care of discharged men has provided a reason and an opportunity we hope that the ward will never be closed again. Any other institution which has been misguided enough to close a ward in the past should seize the opportunity as the David Lewis Northern Hospital has done.

### "WRENS" IN HOSPITAL.

The loss of life among the members of the W.A.A.C. in France which has been announced may remind us that the women in the Services at home also require hospital treatment. Thus the Royal Portsmouth Hospital has agreed to treat members of the "Wren" Corps, who are at work at the naval barracks, at 5s. per day. In response to a request from the medical officer in charge of the W.A.A.C. at the Clarence Barracks, the committee of the hospital has agreed to admit emergency cases arising in that corps on similar terms.

### THE MERTHYR VALE MINERS' DECISION.

By ballot the coal-miners of Merthyr Vale have decided to increase by one penny per man per week during the war and for six months afterwards their hospital levy on behalf of Merthyr and Cardiff institutions. We hope that their generous example will be followed elsewhere.

### THE TREATMENT OF TUBERCULOSIS IN PETERBOROUGH.

The report of the Tuberculosis Officer for the Soke of Peterborough, for the year ended upon August 31, 1917, contains a record of good work upon a small scale in a compact area. The cases of pulmonary tuberculosis are divided in the usual way into three degrees according to the prognostic opinion formed concerning them. Of fifty-eight patients regarded as being in the first stage of disease "forty-three are at work, and in thirty-six of these the disease can be considered arrested." These immediate results are satisfactory and encouraging, more especially to a lay committee. The fact, however, must be insisted upon that statistics of tuberculosis treatment are of value in proportion to the length of time over which observation of patients extends. We should be glad to see a general adoption of uniform tabular statements showing the condition of patients at the end, say, of each yearly period from the time at which they first came under notice or under treatment. From such information only, laborious and slow though it be to obtain, is it possible to gain a knowledge of the average duration of life, or of working capacity of patients after treatment? Controversies as to the comparative value of sanatorium treatment and of the methods of its application can be settled only upon the basis of ultimate results. These often show the futility of the brief periods of treatment which may produce such striking seeming improvement in a patient's health; an improvement which is not a restoration and is as temporary as it is delusive and disappointing. The report quotes cases showing excellent results from treatment by the production of artificial pneumothorax, especially in those in which recurrent hæmorrhages had occurred; stress is laid upon the value of this mode of treatment as one of the few available and useful in advanced cases. No reference is made to any provision of facilities for "hospital" segregation.

### POOR-LAW WOMEN VISITORS.

The Advisory Committee of the Birmingham Board of Guardians has prepared its report upon the position which has arisen from the resignation of Miss Hayward and six women visitors. The suggestion that all the women visitors should be placed under the direct control of the superintendent relieving officer has been rejected. The committee has come to the following conclusions: (1) That the Board appoint a chief woman visitor with salary, and (2) six women visitors, salary commencing at £90, and advancing to the present maximum of £110 plus war bonus, and that where necessary the present visitors be brought up to that scale; and (3) that, pending the appointment of a chief woman visitor, temporary arrangements be made.

### THE SALARIES OF A MUNICIPAL HOSPITAL STAFF.

Willesden Council has considered the question of staffing at the municipal hospital, and appointed Dr. Mabel Campbell Clarke junior resident medical officer at a salary of £300 per annum. The medical officer has been instructed to make arrangements for the necessary consultant staff. Two sisters have been appointed at salaries from £50 to £56 per annum, with a £4 war bonus, and the present sisters are to be paid at this rate. A night sister is to be appointed at a salary of £60, with £4 war bonus. The domestics, having asked for an increased war bonus, are to have £4 instead of £2.

### BITTER CRY OF THE HONEY-LOVER.

Honey is practically the only sweetening agent that is not under official control, and the result is that the selling value has risen to fabulous figures. This has caused great dismay among honey-lovers. "Should our ships," writes a correspondent, "be used at a time like this, and men's lives be risked, to fetch produce across the seas for the sole benefit of the man who is prepared to outbid his neighbour? Should hospitals be made to pay through the nose for honey because the price has been run up by the fashionable confectioner? If the answers to these questions are in the affirmative, then let the Food Controller keep his hands off honey; let the war profiteer have the good things of the earth to himself." We sympathise with our correspondent, who perhaps has an unusually sweet tooth; but we doubt whether the amount of honey imported is a serious item of tonnage. The price of honey probably goes up because of the relative scarcity of jam and marmalade.

### BLUE SPIRITS.

In future methylated spirits will be coloured violet. The reason is that available supplies of wood naphtha are much reduced, and in order to conserve them a smaller quantity of wood naphtha must be used to make methylated spirit. In future 5 parts of wood naphtha and $\frac{1}{2}$ part of mineral naphtha will be added to 95 parts of spirit, and the whole coloured with methyl violet. The mixture will still be undrinkable, and the colour will make it still less inviting. Hitherto ordinary methylated spirit has contained 10 per cent. of wood naphtha and $\frac{3}{8}$ per cent. of mineral naphtha. Industrial methylated spirit will in future contain 2 per cent. of wood naphtha and $\frac{1}{2}$ per cent. of mineral naphtha; hitherto it has contained 5 per cent. of wood naphtha and no mineral naphtha. In processes where the presence of mineral naphtha is altogether unsuitable, the Surveyor of Customs and Excise has power to grant permission for the use, subject to certain restrictions, of a special spirit containing 3 per cent. of wood naphtha and no mineral naphtha.

### THIS WEEK'S DRUG MARKET.

The output of British-made saccharin is increasing, and it now seems probable that before long it will be sufficient to meet even the present heavy demand, and that we shall be able to do without imported saccharin. Supplies of bromides are expected to arrive from the United States shortly, and it is reported that the British Government intends to fix the selling price; in view of this buyers are proceeding cautiously since the fixed prices will probably be below those at present ruling; under the circumstances it would seem wise to purchase according to immediate requirements only. The rumour that the Food Controller intends to fix maximum prices of honey has depressed the market, and buyers are not disposed to operate except at a reduced figure; large quantities —or what would be considered large in normal times—are available. Potassium permanganate is offered more freely, and the price shows signs of receding. Almond oil is again dearer. Business has been done in Curaçoa aloes, sarsaparilla, and gum benzoin at higher prices. Acetanilide and hexamine continue to be in short supply, and prices are firmly maintained. In view of the difficulty of obtaining considerable supplies of Norwegian cod-liver oil it is worthy of note that the output of refined Newfoundland oil (in proportion to the output of common oil) is increasing each season.

### TO OUR READERS.

Contributions are specially invited from any of our readers to these columns. They should deal with topical subjects and news. They must be authenticated for the information of the Editor only. The minimum payment if published is 5s. There is no hard-and-fast rule as to space, but notes of about twenty lines in length are preferred.

## Figures and Tables

**Figure f1:**